# The mixed liver and kidney transcriptome dataset of *Darevskia valentini* rock lizard

**DOI:** 10.1186/s13104-022-06228-4

**Published:** 2022-11-08

**Authors:** Sergei S. Ryakhovsky, Daria V. Zhernakova, Vitaly I. Korchagin, Andrey A. Vergun, Anastasiya E. Girnyk, Victoria A. Dikaya, Marine S. Arakelyan, Aleksey S. Komissarov, Alexey P. Ryskov

**Affiliations:** 1 Laboratory of Genome Organization, Institute of Gene Biology of the Russian Academy of Sciences, 34/5 Vavilova Str., 119334 Moscow, Russia; 2 Applied Genomics Laboratory, SCAMT Institute, ITMO University, 9 Lomonosova Str, 197101 Saint Petersburg, Russia; 3 Laboratory of Genomic Diversity, Center for Computer Technologies, ITMO University, 49 Kronverksky Ave., 197101 Saint Petersburg, Russia; 4 Department of Biochemistry, Molecular biology and Genetics, Moscow Pedagogical State University, 1/1 M.Pirogovskaya Str, 119991 Moscow, Russia; 5 Laboratory of Non-Coding DNA, Institute of Cytology of the Russian Academy of Sciences, 4 Tikhoretsky Ave. , 194064 Saint-Petersburg, Russia; 6 Faculty of Biology, Yerevan State University, 1 Alex Manoogian, 0025 Yerevan, Armenia

**Keywords:** Caucasian rock lizards, genus *Darevskia*, interspecific hybridization, parthenogenesis, *Darevskia valentini*, transcriptome assembly

## Abstract

**Objectives:**

This study is performed in the frame of a bigger study dedicated to genomics and transcriptomics of parthenogenesis in vertebrates. Among vertebrates, obligate parthenogenesis was first described in the lizards of the genus *Darevskia.* In this genus, all found parthenogenetic species originated via interspecific hybridization. It remains unknown which genetic or genomic factors play a key role in the generation of parthenogenetic organisms. Comparative genomic and transcriptomic analysis of parthenogens and their parental species may elucidate this problem. *Darevskia valentini* is a paternal species for four (of seven) parthenogens of this genus, which we promote as a particularly important species for the generation of parthenogenetic forms.

**Data description:**

Total cellular RNA was isolated from kidney and liver tissues using the standard Trizol Tissue RNA Extraction protocol. Sequencing of transcriptome libraries prepared by random fragmentation of cDNA samples was performed on an Illumina HiSeq2500. Obtained raw sequences contained 117,6 million reads with the GC content of 47%. After preprocessing, raw data was assembled by Trinity and produced 491,482 contigs.

## Objective

Hybrid speciation can be considered one of the main variants of reticulate evolution [1]. In some cases, this phenomenon results in the formation of clonal lineages and parthenogenetic species. This study is performed in the frame of a bigger study dedicated to genomics and transcriptomics of parthenogenesis in vertebrates. Till now we carried out for the first time whole-genome sequencing and assembly of trio lizard species, parthenogenetic *Darevskia unisexualis*, and its parental species *D. valentini* and *D. raddei*. However, these data were not published because genome annotations were not yet done. Among vertebrates, obligate parthenogenesis was first described in the rock lizards of the genus *Darevskia* [2], which include 29 bisexual and seven unisexual (parthenogenetic) species, distributed in the Caucasus region, Turkey, and Iran [3]. In this genus, as in most known instances, all found parthenogenetic species originated via interspecific hybridization between closely related bisexual species [4]. Distinctive features of the *Darevskia* rock lizards are the high diversity of parthenogens (seven diploid forms) and ongoing hybridization events in sympatry zones of sexual and parthenogenetic species resulting in triploid and tetraploid hybrids which are considered an intermediate stage of reticulate evolution [5]. The origin of *Darevskia* parthenogens is phylogenetically constrained [6]. Only four parental bisexual species are involved in the origin of seven parthenogens: *D. valentini* and *D*. *portschinskii* as the paternal species and *D. raddei* and *D. mixta* as the maternal species [6, 7]. It remains unknown, which genetic or genomic factors play a key role in the generation of clonally reproduced parthenogenetic organisms. Comparative genomic and transcriptomic analysis of parthenogens and their parental species may elucidate this problem. In particular, *Darevskia valentini* is a paternal species for four (of seven) *Darevskia* parthenogens, that we promote as a particularly important species for the generation of parthenogenetic forms.

## Data description

Samples of *D. valentini* for transcriptome analysis were collected in Armenia in 2016, outside of the protected areas. All individuals were hand-caught. A single adult lizard of male *D. valentini* from the gorge population near the Sepasar village (41°01’39.2"N, 43°48’58.0"E) was used to surgically extract organs (liver, kidneys). Before dissecting the organs, the animals were subjected to chloroform euthanasia followed by decapitation. All tissue samples were stored in RNAlater® reagent at − 20 °C according to the manufacturer’s recommended protocol (Qiagen Inc.) until they were shipped to Macrogen Inc. (Korea) for RNA extraction and further transcriptome sequencing.

**Table 1:** Overview of data files/datasets

Label	Name of data file/data set	File types (file extension)	Data repository and identifier (DOI or accession number)
*Data set 1*	*Raw RNA reads*	*Fastq files (.fastq)*	*NCBI Sequence Read Archive*https://identifiers.org/ncbi/insdc.sra:SRX14421363 [11]
*Data file 1*	*Summary of raw RNA and assembly characteristics*	*Microsoft Word file (.docx)*	*figshare*10.6084/m9.figshare.17762030 [13]
*Data file 2*	*De novo assembly by Trinity*	*Fasta file (.fasta)*	*NCBI Transcriptome Shotgun Assembly Sequence Database*https://identifiers.org/nucleotide:GJZU00000000.1 [14]
*Data file 3*	*TransDecoder peptides*	*Peptide file (.pep)*	*figshare*10.6084/m9.figshare.17696930 [17]
*Data file 4*	*BLASTp, PFAM, EggNOG proteins*	*Table (.csv)*	*figshare*10.6084/m9.figshare.17696915.v2 [19]
*Data file 5*	*Top GO terms*	*Compressed PDF files(.zip)*	*figshare*10.6084/m9.figshare.17696939 [20]
*Data file 6*	*Summary of Trinotate, TransDecoder and top blasted species*	*Compressed text, Excel and pdf files(.txt, .xls, .pdf)*	*figshare*10.6084/m9.figshare.17696951 [21]

Total RNA was isolated from an organs/tissues using standard Trizol Tissue RNA Extraction protocol and was used to prepare the cDNA library The paired-end sequencing libraries were prepared by random fragmentation of the cDNA samples into 350–500 bp fragments, followed by 5’ and 3’ adapter ligation using TruSeq RNA Sample Prep Kit v2 (Illumina Inc.) according to TruSeq RNA Sample Preparation Guide (Version 2, Part #15,026,495 Rev.F). Sequencing of transcriptome libraries was performed on Illumina HiSeq2500 with a mean read length of 101 bp. The Illumina Hiseq generated raw sequencing data utilizing HiSeq Control Software v2.2 for system control and base calling through an integrated primary analysis software. The BCL (base calls) binaries were converted into FASTQ format by the Illumina package bcl2fastq v1.8.4 [8] (RRID:SCR_015058). Raw transcriptome data were trimmed by Trimmomatic v0.39 to remove adapters and deduplicated by the rmdup tool [9, 10] (Data set 1) [11]. Filtered reads quality was assembled using Trinity v2.1.1 [12] with the default minimum contig length value and k-mer size parameters of 200 and 25, respectively. Summary statistics of raw samples, reads, and assembly can be accessed in Data file 1 [13]. The assembly contained 491,482 contigs with a median contig length of 923 bp (Data file 2) [14].

The annotation was provided using TransPi v1.1.0-rc pipeline [15] with OnlyAnn (only annotation) mode [16]. This option included such instruments as TransDecoder, and Trinotate. The TransDecoder program was used to predict translated proteins (Data file 3) [17]. EggNog v2.0.1 [18] was used to cross protein sequences with the Gene Ontology database. BLASTp, PFAM, and EggNOG searching tools revealed 26,812, 6496, and 15,399 proteins respectively (Data file 4) [19]. The most significant Gene Ontology terms were identified and visualized by Trinotate (Data file 5) [20]. In cellular components ontology, the nucleus and cytoplasm were dominated. The regulation of transcription of RNA polymerase II was the most over-represented category in biological processes. In molecular functions, the prevailed number of enriched genes was related to the metal ion and ATP binding. The data of top blasted species and full statistics of GO, ORF prediction numbers, and Trinotate full annotation was also performed (Data file 6) [21].

## Limitations

While our transcriptome data can be used for annotation or verification of protein-coding genes in the lizard genome of *D. valentini* and related lizard species, some limitations are connected with a restricted number of tissues (only liver and kidney) taken for generation of the mixed transcriptome.

## Data Availability

The raw data described in this Data note can be freely and openly accessed on the NCBI SRA database under accession ID SRX14421363. Please see Table 1 for details and links to the rest of the data [11, 13, 14, 17, 19–21].

## Abbreviations

cDNA: complementary deoxyribonucleic acid
RNA: ribonucleic acid
BCL: binary base calls
bp: base pair
GO: Gene Ontology
ORF: open reading frame
ATP: adenosine triphosphate
